# Quantitative proteomic sequencing of *F*_**1**_ hybrid populations reveals the function of sorbitol in apple resistance to *Botryosphaeria dothidea*

**DOI:** 10.1093/hr/uhac115

**Published:** 2022-05-17

**Authors:** Xiaowen He, Hui Meng, Haibo Wang, Ping He, Yuansheng Chang, Sen Wang, Chuanzeng Wang, Linguang Li, Chen Wang

**Affiliations:** Shandong Institute of Pomology, Taian, Shandong 271000, China; State Key Laboratory of Crop Biology, College of Life Sciences, Shandong Agricultural University, Taian, Shandong 271018, China; Shandong Institute of Pomology, Taian, Shandong 271000, China; Shandong Institute of Pomology, Taian, Shandong 271000, China; Shandong Institute of Pomology, Taian, Shandong 271000, China; Shandong Institute of Pomology, Taian, Shandong 271000, China; Shandong Academy of Agricultural Sciences, Jinan, Shandong 250100, China; Shandong Institute of Pomology, Taian, Shandong 271000, China; State Key Laboratory of Crop Biology, College of Life Sciences, Shandong Agricultural University, Taian, Shandong 271018, China

## Abstract

Apple ring rot, which is caused by *Botryosphaeria dothidea*, is one of the most devastating diseases of apple. However, the lack of a known molecular resistance mechanism limits the development of resistance breeding. Here, the ‘Golden Delicious’ and ‘Fuji Nagafu No. 2’ apple cultivars were crossed, and a population of 194 *F*_1_ individuals was generated. The hybrids were divided into five categories according to their differences in *B. dothidea* resistance during three consecutive years. Quantitative proteomic sequencing was performed to analyze the molecular mechanism of the apple response to *B. dothidea* infection. Hierarchical clustering and weighted gene coexpression network analysis revealed that photosynthesis was significantly correlated with the resistance of apple to *B. dothidea*. The level of chlorophyll fluorescence in apple functional leaves increased progressively as the level of disease resistance improved. However, the content of soluble sugar decreased with the improvement of disease resistance. Further research revealed that sorbitol, the primary photosynthetic product, played major roles in apple resistance to *B. dothidea*. Increasing the content of sorbitol by overexpressing *MdS6PDH1* dramatically enhanced resistance of apple calli to *B. dothidea* by activating the expression of salicylic acid signaling pathway-related genes. However, decreasing the content of sorbitol by silencing *MdS6PDH1* showed the opposite phenotype. Furthermore, exogenous sorbitol treatment partially restored the resistance of *MdS6PDH1*-RNAi lines to *B. dothidea*. Taken together, these findings reveal that sorbitol is an important metabolite that regulates the resistance of apple to *B. dothidea* and offer new insights into the mechanism of plant resistance to pathogens.

## Introduction

Apple is one of the most important agricultural fruit crop species. As a major source of nutrients and antioxidants essential to a healthy diet, apple plays an important role in the global economy and is consumed worldwide. However, during growth and development, there are many stress factors that threaten apple quality and yields worldwide, among which biotic stresses are critical [1]. The improvement of the resistance of apple to biotic stresses has been a target of apple breeding in recent decades [2–4]. Apple ring rot, also known as rough bark disease, is a devastating disease of apple and is one of the greatest challenges in the main apple-producing areas. The pathogen causing apple ring rot is *Botryosphaeria dothidea* [5, 6]. This pathogen harms apple tree branches and fruits, resulting in dry rot disease, withering of new shoots, and fruit decay, ultimately causing a decline in tree production potential and even tree death [5]. Under high-temperature and high-humidity conditions in central China (e.g. Henan, Shanxi) and the eastern Bohai Bay area (e.g. Liaoning, Shandong, Hebei), the rate of orchard loss caused by apple ring rot has been reported to be 30–50%, which seriously restricts the healthy and sustainable development of the apple industry [5, 7].

In recent years, the molecular mechanism of resistance to apple ring rot has attracted extensive amounts of attention. When the accumulation of salicylic acid (SA) is induced by pathogens, SA is mainly synthesized via the isochorismate synthase (ICS) pathway [8]. The resistance of apple to *B. dothidea* is regulated by *MdICS1* through the SA biosynthesis pathway. With transient transgenic overexpression of *MdICS1*, apple fruits show increased resistance to *B. dothidea* compared with controls, whereas the suppression of *MdICS1* expression decreases apple resistance to *B. dothidea* [9]. Both the SA content and the expression of SA-related genes increase upon increased expression of *MdICS1* and decrease upon decreased expression of *MdICS1* [9]. The SNARE protein syntaxin 121 (SYP121) plays a major role in apple resistance to *B. dothidea*. After silencing *MdSYP121* in apple calli, the resistance of *MdSYP121*-silenced apple calli was found to be increased through regulation of oxidation–reduction processes and the SA signaling pathway [10]. *MdMYB73*, an apple R2R3-MYB gene, positively regulates resistance to *B. dothidea*. After *B. dothidea* inoculation, *MdMYB73* is strongly induced in apple fruits. Overexpression of *MdMYB73* in transgenic apple calli enhances resistance to *B. dothidea*, which is accompanied by an increase in the expression levels of SA-related genes and the SA content in transgenic plant materials [11]. These studies provided new insights into the resistance of apple to *B. dothidea* and revealed candidate genes. However, the mechanism of resistance to apple ring rot is still unclear, especially in apple tree branches.

Photosynthesis is the most important biological process in plants, as it is responsible for the synthesis of organic substances and provides energy for ecosystems. Recent studies have revealed that biotic stresses have significant effects on the enzymes required for photosynthesis in plants, suggesting that photosynthesis may be closely related to disease resistance. Wheat kinase START1 (WKS1) confers broad-spectrum resistance to *Puccinia striiformis* f. sp. *tritici* (*Pst*) races, which is accompanied by leaf chlorosis [12]. The most recent study on this topic demonstrated that PsbO (an extrinsic member of PSII) is phosphorylated by WKS1, decreasing the binding affinity of PsbO for the PSII core complex and promoting PsbO degradation. Changes in PsbO activity negatively affect the photosynthesis rate and generate an unfavorable environment for *Pst* growth [13]. In rice, LHCB5, a light-harvesting complex II protein, is subject to light-induced phosphorylation during *Magnaporthe oryzae* infection. The phosphorylation of LHCB5 increases the accumulation of reactive oxygen species and provides broad-spectrum resistance of rice to *M. oryzae* [14]. Although the relationship between photosynthesis and disease resistance has been reported, the role of photosynthesis in regulating plant disease resistance is unclear. Sorbitol is a primary photosynthetic product of Rosaceae plants [15, 16]. The metabolism and transport of sorbitol play important roles not only in the accumulation of sugar in fruit and the maintenance of high photosynthesis rates in source leaves but also in regulating pollen tube growth, stamen development and resistance to *Alternaria alternata* in apple [17]. In the cytosol of source leaves, aldose-6-phosphate reductase (A6PR) [also referred to as NADP-sorbitol-6-phosphate dehydrogenase (S6PDH)] and sorbitol-6-phosphate phosphatase are two key enzymes involved in sorbitol synthesis. A6PR is mainly responsible for converting glucose-6-phosphate to sorbitol-6-phosphate, and sorbitol-6-phosphate is then dephosphorylated by sorbitol-6-phosphate phosphatase to yield sorbitol. Previous studies have shown that using antisense inhibition to suppress *A6PR* expression in ‘Greensleeves’ apple leaves significantly decreases sorbitol synthesis and sorbitol levels [15]. Decreasing the level of the *A6PR* transcript leads to the downregulation of the expression levels of 56 nucleotide-binding/leucine-rich repeat (NLR) genes and switches the phenotypic response to *A. alternata* from resistant to susceptible in transgenic plants [17]. These results show that sorbitol may be the link between photosynthesis and the immune response in Rosaceae plants. However, the mechanism by which sorbitol affects plant resistance to other fungi is still unclear.

In China, apple is the most popular fruit, and the apple industry is a pillar industry for regional economic development and farmer income growth. ‘Fuji’ is the main variety produced in the country. However, ‘Fuji’ plants are highly susceptible to apple ring rot, which severely limits the development of the apple industry. ‘Golden Delicious’ is another widely cultivated variety. Although the quality of ‘Golden Delicious’ is inferior to that of ‘Fuji’, it exhibits higher resistance to apple ring rot. To breed high-quality disease-resistant cultivars, our group constructed *F*_1_ hybrid populations from ‘Golden Delicious’ crossed with ‘Nagafu No. 2’ and obtained 277 hybrid seeds. After stratification and sowing propagation, a total of 194 *F*_1_ hybrid seedlings were obtained in 2010. In this work, through the evaluation of disease resistance to apple ring rot, the different disease resistance levels of *F*_1_ progeny were determined. A proteomics analysis of the response of *F*_1_ hybrids to *B. dothidea* infection was performed using hierarchical clustering and weighted gene coexpression network analysis (WGCNA). Combined with the measurement of chlorophyll fluorescence and the expression of photosynthesis-related genes, the results indicated that the *F*_1_ hybrids with high resistance to apple ring rot also showed high photosynthesis levels. The results from a series of genetic, transgenic, and transmission electron microscopy (TEM) experiments suggested that sorbitol was an important metabolite to stimulate the SA-mediated signaling pathway by regulating the expression of SA-related genes and improving the resistance of apple to *B. dothidea*. Our collective findings defined the relationship between photosynthesis and apple resistance to *B. dothidea* and provided theoretical evidence for breeding new varieties tolerant to apple ring rot.

## Results

### The disease resistance levels and protein profiles of *F*_1_ progeny of the ‘Golden Delicious’ and ‘Fuji Nagafu No. 2’ apple varieties

**Figure 1:**
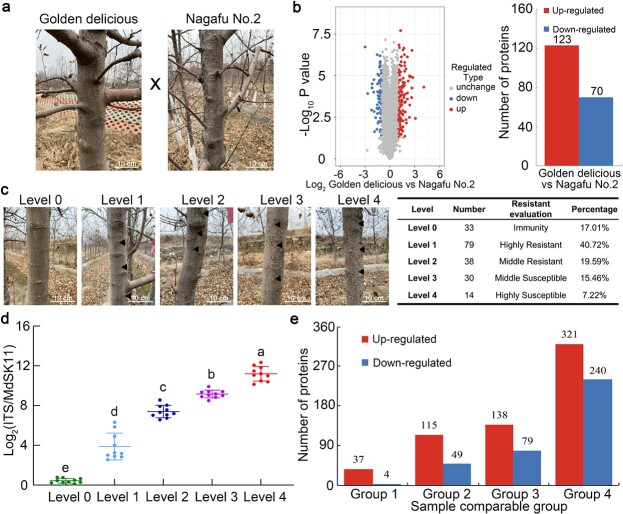
Apple ring rot symptoms and differentially expressed proteins in ‘Golden Delicious’, ‘Nagafu No. 2’ and *F*_1_ hybrid populations. **a** Representative phenotypes of ‘Golden Delicious’ and ‘Nagafu No. 2’ planted in the field. **b** Number of DEPs between ‘Golden Delicious’ and ‘Nagafu No. 2’ based on proteomics. **c** Representative phenotypes and classification of *F*_1_ progeny with different levels of *B. dothidea* resistance (level 0 to level 4). Black arrowheads indicate lesion locations. **d** Pathogen disease index *F*_1_ progeny with different levels of *B. dothidea* resistance (level 0 to level 4). Error bars indicate the mean ± standard error of three independent experiments (*n* = 10). Different letters indicate significant differences (*P* < .01) based on Tukey’s HSD test. **e** Numbers of DEPs identified between group 1 (level 1 versus level 0), group 2 (level 2 versus level 0), group 3 (level 3 versus level 0), and group 4 (level 4 versus level 0).

In this research, a population of 194 *F*_1_ individuals derived from a cross between the resistant variety ‘Golden Delicious’ and ‘Fuji Nagafu No. 2’ was obtained to identify new genotypes with high quality and resistance (Fig. 1a). The hybrids were divided into five categories according to their differences in *B. dothidea* resistance during three consecutive years (2016–18): level 0 (immune); level 1 (highly resistant); level 2 (moderately resistant); level 3 (moderately susceptible); and level 4 (highly susceptible) (Fig. 1c). The five categories included 33, 79, 38, 30, and 14 progenies, respectively (Fig. 1c). To verify the rationality of this disease classification approach, the trunk bark tissues of ten independent strains, randomly selected from each category, were used for disease-level determination. The pathogen disease index of the selected samples was measured, and the *B. dothidea* content increased with decreasing degree of disease resistance (Fig. 1d).

To explore the molecular mechanisms of apple resistance to *B. dothidea*, we combined a series of technologies, including tandem mass tag (TMT) labeling, HPLC fractionation and LC–MS/MS analysis, to perform a quantitative analysis of the global proteomes of the bark from maternal (‘Golden Delicious’) and paternal (‘Fuji Nagafu No. 2’) parents and the *F*_1_ progeny with different levels of *B. dothidea* resistance (level 0 to level 4). Altogether, 8836 proteins were identified, 8229 of which were quantified (Supplementary Data Table S1). Three statistical analysis methods were used to evaluate the repeatability of protein quantification (Supplementary Data Fig. S1). First, we compared the differentially expressed proteins (DEPs) between ‘Golden Delicious’ and ‘Nagafu No. 2’. The criterion for up- or downregulation was a fold change >2 or <1/2; here, 123 proteins were upregulated, while 70 proteins were downregulated (Fig. 1b; Supplementary Data Table S2). As the degree of *B. dothidea* resistance decreased, the number of DEPs increased gradually. Additionally, 41, 164, 217, and 561 DEPs were identified in the comparisons of level 1 versus level 0 (group 1), level 2 versus level 0 (group 2), level 3 versus level 0 (group 3), and level 4 versus level 0 (group 4), respectively (Fig. 1e; Supplementary Data Tables S3–S6). Among these significantly differentially expressed proteins, 37, 115, 138, and 321 were upregulated, and 4, 49, 79, and 240 were downregulated in different *F*_1_ hybrid population groups (Fig. 1e).

**Figure 2:**
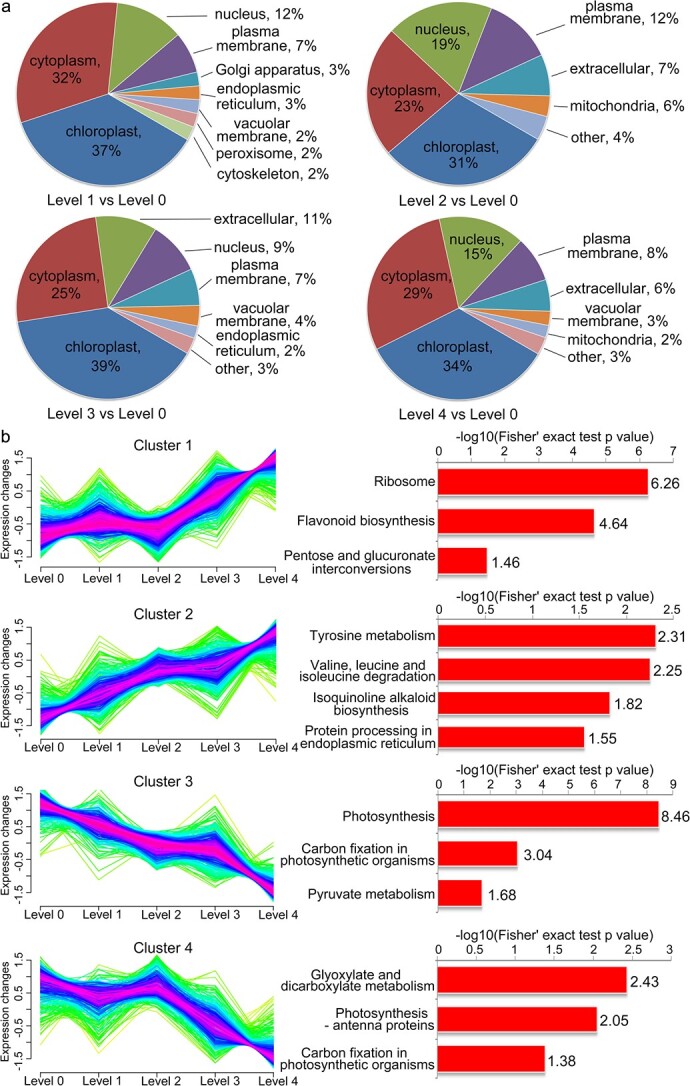
Subcellular localization and hierarchical clustering analysis of DEPs in *F*_1_ progeny. **a** Subcellular localization of DEPs according to GO (Gene Ontogeny) analysis in different groups. **b** Hierarchical clustering and KEGG enrichment analysis of the DEPs. For each dataset, four major clusters were identified (left), each of which was subjected to KEGG enrichment analysis (right).

To characterize the subcellular localization with different levels of resistance to *B. dothidea*, prediction of the subcellular localizations of DEPs was performed (Fig. 2a). In the subcellular classification analysis, the chloroplast accounted for the largest proportion of DEPs, followed by the cytoplasm in the different *F*_1_ hybrid population groups, and the percentage of proteins located in these subcellular structures was similar among the different groups. These results implied that chloroplasts might be key organelles involved in the regulation of apple resistance to *B. dothidea* (Fig. 2a).

### Functional enrichment analysis of differentially expressed proteins

To explain the biological functions of the DEPs in apple resistance to *B. dothidea*, hierarchical clustering and KEGG (Kyoto Encyclopedia of Genes and Genomes) enrichment analysis were performed to assess protein coexpression (Fig. 2b). We extended our analysis via a ‘guilty-by-association’ approach. The hierarchical clustering analysis of proteomic abundance revealed disease resistance regulation in the apple proteome. The proteins with the same expression trend were selected from all the DEPs to form a cluster. Four major clusters were identified: cluster 1, consisting of 565 proteins, mainly related to the function of ribosomes; cluster 2, consisting of 451 proteins, focused on amino acid metabolism; and clusters 3 and 4, consisting of 450 and 402 proteins, respectively, most of which showed mixed patterns of abundance related to photosynthesis and carbon fixation (Supplementary Data Table S7). Expression clustering revealed distinct groups of proteins responding to the *B. dothidea* machinery to form highly conserved functional modules (Fig. 2b; Supplementary Data Table S8). One of the groups comprised ribosome (mdm03010), flavonoid biosynthesis (mdm00941) and pentose and glucuronate interconversions (mdm00040) (Fig. 2b and Supplementary Data Table S8, cluster 1). The second predicted protein cluster was related to amino acid metabolism (Fig. 2b and Supplementary Data Table S8, cluster 2). KEGG enrichment analysis revealed that tyrosine metabolism (mdm00350) and valine, leucine, and isoleucine degradation (mdm00280) were mainly involved in apple disease resistance (Fig. 2b and Supplementary Data Table S8, cluster 2). In clusters 3 and 4, the protein expression level was positively correlated with apple resistance to *B. dothidea*. These proteins were most closely associated with photosynthesis (mdm00195) and carbon fixation in photosynthetic organisms (mdm00710) (Supplementary Data Table S8). These results indicated that photosynthesis may play a key role in apple resistance to *B. dothidea*.

### Weighted gene coexpression network analysis identified novel modules associated with the apple response to *B. dothidea*

**Figure 3:**
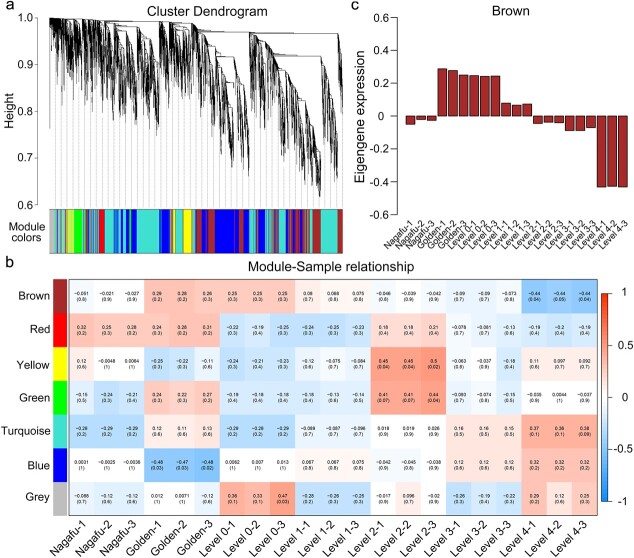
WGCNA of *B. dothidea*-responsive proteins in ‘Golden Delicious’, ‘Nagafu No. 2’ and *F*_1_ hybrid populations. **a** Clustering dendrograms of proteins showing dissimilarity, together with assigned module colors. **b** Module-specimen associations. Each column represents a specimen and each row represents a module eigengene. Numbers in the boxes are the corresponding correlations and *P* values. The *P* values between pairs of modules were calculated by Student’s *t*-test. 1 indicates a positive correlation and −1 indicates a negative correlation. **c** The histogram shows the variation in brown module eigengenes expressed in different samples.

With the availability of large-scale proteome datasets, WGCNA has allowed the identification of a cohort of genes that show similar expression patterns in response to biotic stress. To determine disease-responsive common protein signatures, WGCNA was performed. To construct the coexpression module network regulating the resistance of apple to *B. dothidea*, we processed the expression values of 8836 proteins in 21 samples and removed all the outliers. Correlation coefficients were calculated among the proteins. As shown in Fig. 3a, protein modules were established according to the clustering relationships between proteins. Based on the module feature values, modules with similar expression patterns were then merged. Seven distinct protein coexpression modules were identified from the apple proteomic sequencing results. These coexpression modules were constructed and are shown in different colors in Fig. 3a. The numbers of proteins in the seven modules are shown in Supplementary Data Table S9. A heat map of the correlations between modules and samples showed that the proteins in the brown module presented a high correlation with the differences in the resistance of the samples to *B. dothidea* (Fig. 3b). Furthermore, the expression patterns of the proteins in the brown module are displayed in a heat map, and the changes in eigengene expression values among different samples (equivalent to the module expression pattern) are presented in a histogram. The proteins in the brown module were highly expressed in ‘Golden Delicious’ and were expressed at low levels in ‘Fuji Nagafu No. 2’ (Fig. 3c). With decreasing resistance to *B. dothidea* in the hybrid progeny, the protein expression levels progressively decreased (Fig. 3c). The WGCNA results showed that the proteins in the brown module might be positively correlated with apple resistance to *B. dothidea*.

**Figure 4:**
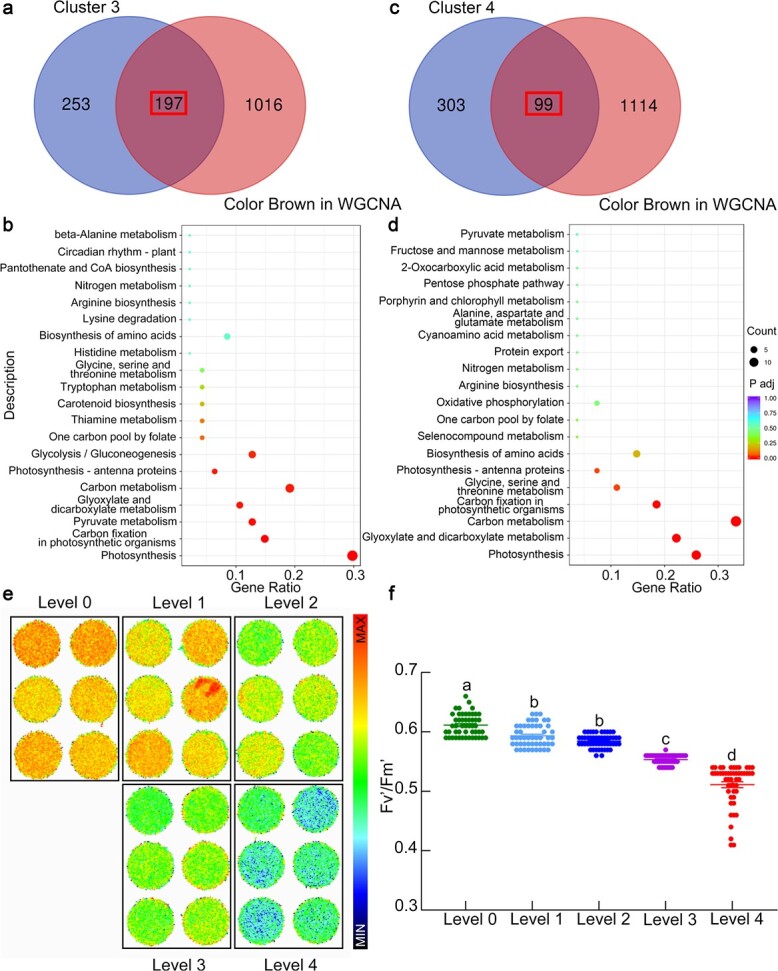
Photosynthesis played important roles in apple resistance to *B. dothidea*. **a** Comparison of the proteins in cluster 3 and the WGCNA brown module. **b** KEGG enrichment analysis of the 197 genes shared by cluster 3 and the brown module in WGCNA. **c** Comparison of the proteins in cluster 4 and the WGCNA brown module. **d** KEGG enrichment analysis of the 99 genes shared by cluster 4 and the brown module in WGCNA. Gene ratio refers to the ratio of the number of DEPs annotated to a KEGG pathway to DEPs annotated to all KEGG pathways. **e**, **f** Chlorophyll fluorescence measured in *F*_1_ hybrid population leaves. Differences in fluorescence (*F*_v_′/*F*_m_′) were observed with a FluoCam FC800 imaging system (Photon Systems Instruments, Czech Republic) at 25°C and 400–420 μmol mol^−1^ CO_2_. Different letters indicate significant differences (*P* < .01) based on Tukey’s HSD test.

To better screen genes closely related to disease resistance, the proteins in cluster 3 and the WGCNA brown module and those in cluster 4 and the WGCNA brown module were considered to be directly involved in disease resistance. As shown in Fig. 4a and Supplementary Data Table S10, 197 proteins were screened by comparing the proteins in cluster 3 and the WGCNA brown module. KEGG enrichment analysis of the 197 proteins revealed that photosynthesis (mdm00195) and carbon fixation in photosynthetic organisms (mdm00710) may play important roles in apple resistance to *B. dothidea* (Fig. 4b). In addition, 99 proteins were screened by comparing cluster 4 with the WGCNA brown module (Fig. 4c; Supplementary Data Table S11). KEGG enrichment analysis of the 99 proteins showed that most proteins were also related to photosynthesis (mdm00195), carbon metabolism (mdm01200), and carbon fixation in photosynthetic organisms (mdm00710) (Fig. 4d). Combined analysis revealed that photosynthesis and carbon metabolism were closely related to resistance to *B. dothidea* in apple.

### Photosynthesis plays important roles in apple resistance to *B. dothidea*

To verify the proteomic results, qRT–PCR was performed to analyze the expression levels of photosynthesis-related genes in different *F*_1_ hybrid populations. Twelve photosynthesis-related genes were selected from the photosynthesis KEGG pathway (KEGG ID mdm00195) based on the 197 genes shared by cluster 3 and the brown module in WGCNA or the 99 genes shared by cluster 4 and the brown module in WGCNA. The expression levels of the 12 photosynthesis-related genes decreased with decreasing disease resistance (Supplementary Data Fig. S2). To further clarify the effect of photosynthesis on apple resistance, chlorophyll fluorescence was measured in 194 *F*_1_ progeny trees. As the level of disease resistance declined, the chlorophyll fluorescence of apple functional leaves decreased, and the excitation energy capture by open PSII reaction centers (*F*_v_′/*F*_m_′), maximum quantum yield of PSII (*F*_v_/*F*_m_), and quantum yield of PSII (*Φ*_PSII_) decreased progressively with the decrease in resistance to *B. dothidea*, reaching the lowest level in highly susceptible apple leaves (Fig. 4e and f; Supplementary Data Fig. S3). Furthermore, to determine whether pathogen infection leads to the decline of photosynthesis or whether a low level of photosynthesis leads to low disease resistance, grafting plants were used to detect the expression level of photosynthesis-related genes and the level of chlorophyll fluorescence. The grafted plants were obtained from the healthy branches of the *F*_1_ populations (selected based on high resistance or high quality). As shown in Supplementary Data Fig. S4 and S5, the healthy grafting plants obtained from level 0 *F*_1_ populations showed higher levels of gene expression and chlorophyll fluorescence. These results suggested that the intensity of photosynthesis was closely related to apple resistance to *B. dothidea*.

**Figure 5:**
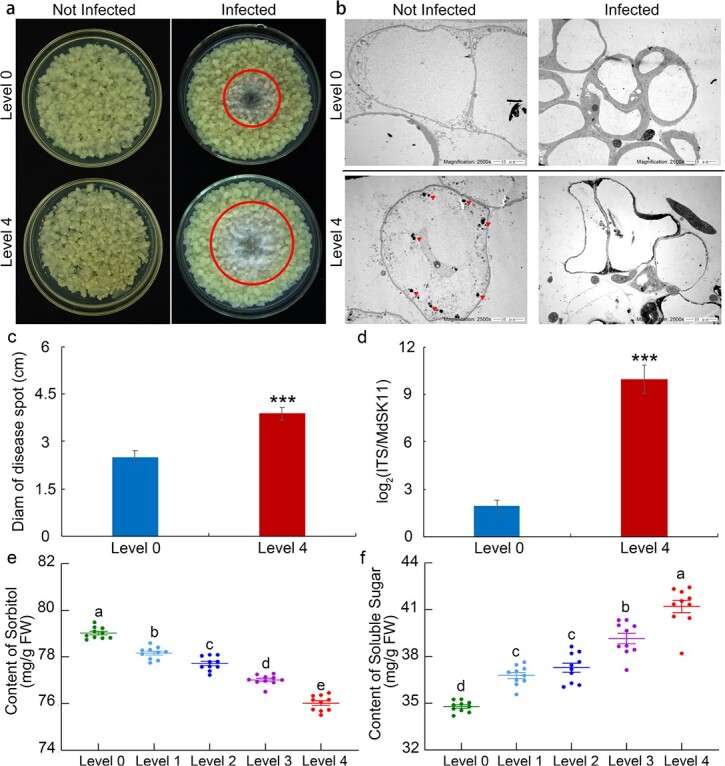
Validation of *F*_1_ hybrid population resistance to *B. dothidea*. **a** Representative phenotypes of calli of highly susceptible progeny (level 4) and immune progeny (level 0) before and after *B. dothidea* infection. **b** Status of apple calli growth and *B. dothidea* infection in level 0 and level 4 calli. Red arrowheads indicate starch granules. **c**, **d** Pathogen disease index in level 0 and level 4 calli infected with *B. dothidea* for 4 days. The data are the means ± standard errors of three independent experiments (*n* = 6). ^***^P < .001 based on Tukey’s HSD test. **e** Content of sorbitol in different *F*_1_ hybrid populations. **f** Content of soluble sugar in different *F*_1_ hybrid populations. Error bars indicate the means ± standard errors of three independent experiments (*n* = 10). Different letters indicate significant differences (*P* < .01) based on Tukey’s HSD test.

To further elucidate the mechanism of apple resistance to *B. dothidea*, apple calli from highly susceptible progeny (level 4) and immune progeny (level 0) were cultured, and there was little difference in the growth trend between the two types of calli without treatment (Fig. 5a). At 4 days after *B. dothidea* inoculation, the disease lesion area in the highly susceptible progeny was significantly larger than that in the immune progeny (Fig. 5a and c). TEM (JEM-1200EX) was used to observe the status of apple calli growth and *B. dothidea* infection. The results showed that a high percentage of starch granule deposition was easily detectable in cytoplasmic structures in the highly susceptible strain, and *B. dothidea* was more harmful to the calli of the highly susceptible progeny (Fig. 5b). The pathogen disease index of the highly susceptible strain calli was much higher than that of the immune strain calli (Fig. 5c and d). Additionally, the contents of sorbitol and soluble sugar were measured in 10 randomly selected samples from each category. With declining disease resistance, the content of sorbitol in the *F*_1_ apple hybrids significantly decreased, while the content of soluble sugar increased (Fig. 5e and f).

### Sorbitol positively regulated apple resistance to *B. dothidea*

**Figure 6:**
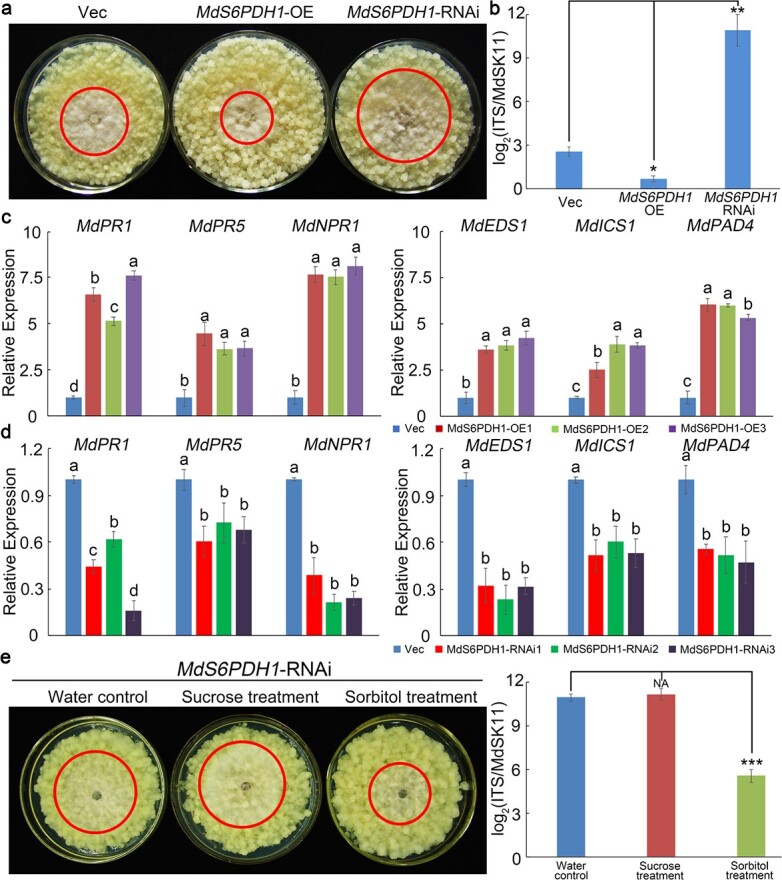
*MdS6PDH1* played important roles in apple resistance to *B. dothidea*. **a** Representative phenotypes of Vec, *MdS6PDH1*-OE, and *MdS6PDH1*-RNAi calli infected with *B. dothidea*. **b** Pathogen disease index in Vec (empty vector control) and transgenic calli infected with *B. dothidea* for 4 days. **c**, **d** qRT–PCR analysis of the expression of the SA signaling pathway marker genes and SA synthesis-related genes in Vec and transgenic calli infected with *B. dothidea*. **e** Representative phenotypes of *MdS6PDH1*-RNAi calli infected with *B. dothidea* after exogenous sorbitol treatment. The error bars indicate the means ± standard errors of three independent experiments (*n* = 9). ^*^P < .05; ^**^P < .01; ^***^P < .001. Different letters indicate significant differences (*P* < .01) based on Tukey’s HSD test.

The above results indicated that photosynthesis was significantly increased in *F*_1_ progenies with high resistance to *B. dothidea*, and the content of sorbitol was also markedly increased. Sorbitol is a major phloem transport carbohydrate and photosynthate in Rosaceae plants. Furthermore, in clusters 3 and 4, the data showed that the expression level of S6PDH [gene ID MdS6PDH1 (MD02G1264100) and MdS6PDH2 (MD05G1054800)], which is the key enzyme in the synthesis of sorbitol and directly affects the interconversion of sorbitol and soluble sugar, showed correspondingly low expression levels as disease resistance decreased in the *F*_1_ generation (Supplementary Data Fig. S6, Supplementary Data Table S7). After *B. dothidea* inoculation for 4 days, the sorbitol content and expression level of *MdS6PDH1* were increased in ‘Orin’ calli (Supplementary Data Fig. S7). These results indicated that sorbitol might play important roles in apple resistance to *B. dothidea*. To explore the function of sorbitol in apple disease resistance, exogenous sorbitol was used to treat level 4 calli. As shown in Supplementary Data Fig. S8, the resistance of level 4 calli to *B. dothidea* was partially restored after exogenous sorbitol (50 mM) treatment. To further clarify the biological function of sorbitol in apple resistance to pathogens, *MdS6PDH1* was silenced or overexpressed in ‘Orin’ calli. For further functional analysis, three independent transgenic lines were selected (Supplementary Data Fig. S9a and c). The content of sorbitol in the *MdS6PDH1*-overexpressing (*MdS6PDH1*-OE) lines was higher than that in the Vec line (empty vector control), while that of the *MdS6PDH1*-silenced calli (*MdS6PDH1*-RNAi) lines was lower (Supplementary Data Fig. S9b and d). Four days after *B. dothidea* inoculation, the *MdS6PDH1*-RNAi lines showed more severe signs of spot extension areas than the Vec line, and the degree of morbidity in *MdS6PDH1*-OE lines was lower than that in the Vec line (Fig. 6a). The pathogen disease index of the *MdS6PDH1*-OE lines was much lower than that of the Vec line, while that of the *MdS6PDH1*-RNAi lines was higher (Fig. 6b). The expression levels of the SA signaling pathway marker genes [*Pathogen resistance* (*PR*) genes (*MdPR1* and *MdPR5*) and *MdNPR1*] [18, 19] and SA synthesis-related genes [*Enhanced disease susceptibility 1* (*MdEDS1*), *Phytoalexin deficient 4* (*MdPAD4*), and *Isochorismate synthase 1* (*MdICS1*)] [20–22] were much higher in the *MdS6PDH1*-OE lines than in the Vec line after *B. dothidea* infection, while the *MdS6PDH1*-RNAi lines showed lower expression (Fig. 6c and d). Exogenous sorbitol (50 mM) was used to treat *MdS6PDH1*-RNAi calli lines. The exogenous sorbitol treatment partially restored the resistance of *MdS6PDH1*-RNAi lines to *B. dothidea* (Fig. 6e). As shown in Supplementary Data Fig. S10, the exogenous sorbitol treatment also increased the content of SA in ‘Orin’ calli. Furthermore, the expression levels of the SA signaling pathway marker genes and SA synthesis-related genes in the leaves of level 4 plants were induced after exogenous sorbitol (50 mM) treatment for 3 h (Supplementary Data Fig. S11). These results suggested that sorbitol positively regulated the resistance of apple to *B. dothidea*.

## Discussion

Apple ring rot, caused by *B. dothidea*, is one of the most devastating diseases of apple and greatly affects apple production. Thus, apple ring rot is a serious problem that remains to be solved in apple cultivation. Sorbitol, a major product of photosynthesis in Rosaceae plants, plays critical roles in multiple biological processes [15, 16, 23]. The function and mechanisms of sorbitol in regulating apple resistance to *B. dothidea* remain unknown. In this study, we revealed that sorbitol functions as a bridge that links photosynthesis and plant immunity, and we established a potentially important function of sorbitol in apple defense against the apple ring rot pathogen.

Proteomic analysis of DEPs is a powerful tool for studying gene function and exploring the molecular mechanisms by which organisms respond to biotic stresses. Protein cluster analysis and WGCNA can be used to accurately identify the key regulatory processes related to a particular phenotypic trait of plants. In rice, proteomics-based protein cluster screening was developed to identify putative small secreted proteins (SSPs) induced by *M. oryzae* and its elicitor chitin. A total of 236 SSPs were identified, and they included members of small signaling peptide families that are well known to be involved in immunity, such as proteinase inhibitors and PR protein families [24]. In cotton, WGCNA has enabled the identification of nine novel modules with 52 hubs of highly connected genes showing similar expression patterns in response to cotton leaf curl disease (CLCuD). The differential regulation of auxin stimulus and cellular localization pathways in response to CLCuD was indicated according to the analysis of these hubs [25]. To breed high-quality, *B. dothidea*-resistant cultivars, our group constructed *F*_1_ hybrid populations from a cross of ‘Golden Delicious’ with ‘Nagafu No. 2’. The disease resistance of the *F*_1_ progeny was distributed in a nearly normal fashion (Fig. 1). Proteomic sequencing combined with a protein cluster analysis and WGCNA of *F*_1_ progeny with differences in resistance to *B. dothidea* indicated that photosynthesis was associated with apple resistance to apple ring rot.

Photosynthesis is an important biological process in plants, and its regulation plays a pivotal role in plant defense against biotic stress [26, 27]. Three important biological processes (photosynthesis, pathogen infection, and plant defense) have been investigated separately for decades [28]. Recent studies have shown that photosynthesis is associated with plant disease resistance. When plants are infected with pathogens, photosynthesis generates ATP and NADPH, and carbohydrates are utilized for the synthesis of antimicrobial compounds and defense-related hormones, such as jasmonic acid, SA, ethylene, and abscisic acid [29]. Chloroplasts are indispensable structures for photosynthesis and are the main source of defense-related signaling molecules or their precursors [29]. Photosynthesis may be influenced by defense-related signals by regulating the expression level of photosynthetic protein genes [29, 30]. In *Arabidopsis*, *Pseudomonas syringae* effectors reprogram chloroplast-targeted gene expression, target chloroplasts, and inhibit photosynthetic CO_2_ assimilation through the disruption of PSII. This activity decreases plant disease resistance by preventing reactive oxygen bursts in chloroplasts [31]. Through a detailed comparative analysis of transcriptional responses in *Arabidopsis* leaves with or without *P. syringae* infection, researchers found that the sustained and rapid suppression of most transcripts encoding photosynthetic components was one of the most prominent microbial-associated molecular pattern-triggered immune responses [32]. *Arabidopsis AtNHR2A* and *AtNHR2B*, two orthologous genes of *Nicotiana benthamiana Nonhost Resistance 2*, are novel components of a chloroplast signaling pathway that activates callose deposition in the cell wall in response to bacterial pathogens [33]. Further studies indicated that biotic stress downregulates the global expression levels of genes that encode components of PSI and PSII reaction centers and several elements of LHCII and ATP synthase [34]. Despite all of these data, functional studies on the positive regulation of resistance to pathogens by photosynthesis are limited. In our research, proteome sequencing analysis revealed that most of the DEPs were involved in the process of photosynthesis in diseased plants (Fig. 4). In addition, chlorophyll fluorescence decreased progressively with decreasing resistance to *B. dothidea* and was lowest in the highly susceptible apple leaves (Fig. 4). Furthermore, the healthy *F*_1_ population of grafting plants obtained from the level 0 *F*_1_ population showed higher levels of photosynthesis-related gene expression and chlorophyll fluorescence than the level 2 and level 4 *F*_1_ plants (Supplementary Data Fig. S4 and S5). These results implied that photosynthetic capacity is positively associated with apple resistance to *B. dothidea*.

In plants, sugars and alcohols are the main products of photosynthesis. Previous studies have shown that sugars play a key role in plant disease resistance [35]. As a carbon source for pathogens, plant sugars have the largest impact on determining the outcome of plant–microbe interactions [14]. Accumulating evidence indicates that some rare sugars can act as signaling molecules to stimulate plant immunity and induce defense-related gene expression [35, 36]. Rice (*Oryza sativa*) overexpressing *PRms*, a maize PR gene, can increase sucrose accumulation and develop resistance to *M. oryzae*, which was confirmed by the exogenous feeding of sucrose to wild-type rice [37]. Galactinol and raffinose function as signals to stimulate plant immunity. In tobacco, the external application of galactinol results in the activation of some well-known defense-related genes, including *PR1a*, *PR1b*, and *NtACS1*, under *Pseudomonas chlororaphis* infection [38]. Sugar alcohols also serve as major photosynthates and transport carbohydrates in many plants [39, 40]. Compared with research on sugars, studies on the function of sugar alcohols in plant disease resistance are limited. In Rosaceae plants, sorbitol plays crucial roles in carbohydrate metabolism for plant growth as the primary photosynthetic product and transports carbohydrates [15, 16, 23]. Li *et al.* [41] observed that transgenic plant leaves with lower sorbitol levels developed brown spots similar to symptoms caused by the *A. alternata* apple pathotype. Decreasing sorbitol synthesis via the antisense suppression of *A6PR* in apple has been shown to lead to the downregulation of 56 NLR genes and decreased resistance to *A. alternata* [17]. In this study, the results revealed that the content of soluble sugar was lower in the highly resistant *F*_1_ apple hybrids, while the content of sorbitol was higher in these plants (Fig. 5). Furthermore, the expression of *MdS6PDH1* (also referred to as *MdA6PR*), an important enzyme in the synthesis of sorbitol, was modulated to regulate the content of sorbitol in apple calli. The *MdS6PDH1*-overexpressing apple calli lines presented increased resistance to *B. dothidea*, whereas the RNAi suppression of *MdS6PDH1* showed decreased resistance to *B. dothidea* (Fig. 6). Exogenous sorbitol treatment partially restored the resistance of *MdS6PDH1*-RNAi lines to *B. dothidea* (Fig. 6). In addition, the expression levels of the SA signaling-related genes and the SA synthesis-related genes were much higher in the *MdS6PDH1*-OE lines than in the Vec and *MdS6PDH1*-RNAi lines (Fig. 6). Furthermore, the content of SA was increased after exogenous sorbitol treatment or *B. dothidea* inoculation (Supplementary Data Fig. S10). These findings indicate that sorbitol might act as an important metabolite to modulate resistance to *B. dothidea* by increasing the SA content and activating the SA signaling pathway in apple.

In conclusion, proteomic analysis showed that apple resistance to *B. dothidea* was associated with photosynthesis. As disease resistance decreased, the abundance of photosynthesis-related proteins significantly decreased in the *F*_1_ apple hybrids. Chlorophyll fluorescence also decreased progressively with decreasing resistance to *B. dothidea* in *F*_1_ apple hybrids. More importantly, further research revealed that sorbitol linked photosynthesis and plant immunity and activated SA-mediated *B. dothidea* resistance. These findings clarify the important role of photosynthesis in apple resistance to apple ring rot and imply that sorbitol is an important metabolite that regulates apple immunity.

## Materials and methods

### Plant materials, fungal strains, and growth conditions

In 2009, a segregating *F*_1_ hybrid population was derived from a cross between the ‘Golden Delicious’ and ‘Fuji Nagafu No. 2’ apple cultivars. In 2010, *F*_1_ hybrid seeds were sown after stratification, and seedlings were maintained at the Tai’an Tianping Lake base of the Shandong Institute of Pomology (117°032′E, 36°225′N) without replicates and rootstock. From 2016 to 2018, ‘Golden Delicious’, ‘Fuji Nagafu No. 2’ and *F*_1_ progenies were assessed for apple ring rot resistance following natural infection in the field over three consecutive years. The quantity of infection spots in each tree was the assessment metric employed. According to the degree of apple ring rot observed in the field, the resistance of the *F*_1_ hybrids was divided into five levels (levels 0–4). The soluble sugars (reducing sugars dissolved in water) were measured by anthrone colorimetry using a Plant Soluble Sugar Content Assay Kit (Solarbio, China) according to the manufacturer’s instructions. Sorbitol can form a blue complex with copper ions in alkaline solution and has a maximum absorption peak at a 655-nm wavelength. The content of sorbitol was detected by this principle using a Sorbitol Content Assay Kit (Solarbio, China) according to the manufacturer’s instructions.

According to intrinsic fruit quality, appearance quality, phenotype, and disease resistance, high-resistance or high-quality hybrid seedlings were selected and grafted onto *M_26_* rootstock. Five single-tree replicates per genotype were maintained using standard horticultural practices. The second leaves from the top of the annual branches (the youngest fully developed leaves) were used for chlorophyll fluorescence measurement. Three independent biological replicates were performed for each experiment.

The leaves from *F*_1_ hybrid plants were used as explants to obtain calli. After washing with flowing water, the leaves were scrubbed clean. Under aseptic conditions, the leaves were treated with 75% ethanol for 20 seconds and surface-sterilized with NaClO (5%) for 20 minutes. Then, the leaves were washed extensively five times with distilled water and dried with sterile filter paper. The disinfected leaves were cut into pieces ~1 cm wide and inoculated onto Murashige and Skoog (MS) medium with 6-benzyladenine (1.0 mg/L) and naphthalene acetic acid (0.1 mg/L). Tissue-cultured calli were subcultured under basic growth conditions at 24 ± 0.5°C for 24 hours in darkness (relative humidity 60–75%) on MS medium [6-BA (0.4 mg/L), 2,4-D (1.5 mg/L), sucrose (30 g/L), and agar (7.5 g/L); pH 5.8–6.0]. For sorbitol treatment, exogenous sorbitol (50 mM) [17] was sprayed on the calli after 12 days of growth. A pathogen infection analysis was performed according to the methods of He *et al.* [10]. The content of SA was detected using an HPLC/MS system according to previous research [42]. Treated calli samples were collected from culture dishes, frozen in liquid nitrogen and stored at −80°C for the follow-up experiment.

### Pathogen biomass assay

The pathogen biomass assay was performed based on the methods of Wang *et al.* [43]. The DNAsecure Plant Kit (Tiangen, China) was used to isolate DNA from apple bark or calli. The numbers of *ITS* and *MdSK11* genome sequences were used to represent the number of *B. dothidea* and apple, respectively. The amount of *B. dothidea* DNA (*ITS*) present relative to the amount of bark or calli DNA (*MdSK11*) was determined via qPCR. Disease index [log_2_ (ITS/MdSK11)] values were determined by subtracting the Ct values of *ITS* from those of *MdSK11*. At least three biological replicates were analyzed for all of the samples.

### Quantitative proteomic sequencing

For quantitative proteomic sequencing, the trunk bark of hybrid strains was sampled after defoliation in the late fall and winter of 2018. The total protein of the maternal (‘Golden Delicious’) and paternal (‘Fuji Nagafu No. 2’) parents and the *F*_1_ progeny was extracted from infected bark, and the total protein of the *F*_1_ progeny was combined based on the different levels of *B. dothidea* resistance (levels 0–4). Seedlings of each level were sampled separately and mixed equally. Each sample was collected three times to perform three independent repetitions.

The samples were ground into a cell powder in liquid nitrogen, and four volumes of lysis buffer [urea (8 M), dithiothreitol (10 mM), Triton 100 (1%), and protease inhibitor cocktail (1%)] were added to the cell powder, followed by ultrasonication three times. The proteins were precipitated with cold 20% trichloroacetic acid for 2 hours at −20°C. After centrifugation at 12 000 × g and 4°C for 5 minutes, the supernatant was discarded. The remaining precipitate was washed with cold acetone three times and redissolved in urea (8 M). Then, a BCA kit (Beyotime, China) was used to measure the protein concentration according to the manufacturer’s instructions.

The protein was digested with trypsin (Promega, USA). The digested peptide was desalted by a Strata-X C18 SPE column (Phenomenex) and dried under vacuum. After reconstitution with Triethylammonium bicarbonate (0.5 M), the digested peptide was processed according to the manufacturer’s protocol of the TMT labeling kit (Thermo Fisher Scientific, USA). The level 0, 1, 2, 3, and 4 samples were labeled with the TMT tags 126, 127C, 128 N, 128 C and 129 N, respectively. In addition, the maternal and paternal parents were labeled with the tags 129 C and 130 N. LC–MS/MS analysis and bioinformatics analysis were performed at Jingjie PTM Biolab (Hangzhou, China). The resulting MS/MS data were processed using the MaxQuant search engine (v. 1.5.2.8). Tandem mass spectra were queried against the *Malus domestica* database from UniProt (*M. domestica* 3750 UP 20190710, https://www.UniProt.org/taxonomy/3750). The confidence level of protein identifications was adjusted to a <1% false discovery rate (FDR), and the minimum score for modified peptides was set to >40.

For WGCNA, an R-based WGCNA package was used to construct the coexpression network [44]. The dendrogram was constructed using the cutreeDynamicTree algorithm [45]. The smallest module included 126 proteins. A weighted correlation threshold of ≥0.85 was set and resulted in the identification of seven modules across the entire network. Cytoscape v. 3.5.1 was used for visualizing and analyzing the weighted coexpression network [46, 47].

### Measurements of chlorophyll fluorescence

To minimize the effect of chlorophyll fluorescence induction kinetics, an improved method was used to measure chlorophyll fluorescence [48]. Six to eight mature disease-free leaves of the new shoot of the apple center stem extension head were washed with water and dried with absorbent paper. The leaves were dark-adapted for 1 hour before the measurement. All treatments were repeated three times. Chlorophyll fluorescence transients with dark-adapted leaves were analyzed at room temperature. The minimum fluorescence (*F*_o_), maximum fluorescence (*F*_m_), steady-state fluorescence (*F*_s_), maximum fluorescence in the light-adapted state (*F*_m_′), and minimum fluorescence in the light-adapted state (*F*_o_′) were then determined with a FluoCam FC800 imaging system (Photon Systems Instruments, Czech Republic) at 25°C under 400–420 μmol mol^−1^ CO_2_.

The following parameters were then calculated [49]: (i) maximum quantum yield of PSII, *F*_v_/*F*_m_ = (*F*_m_ − *F*_o_)/*F*_m_; (ii) excitation energy capture by open PSII reaction centers, *F*_v_′/*F*_m_′ = (*F*_m_′ − *F*_o_’)/*F*_m_′; (iii) quantum yield of PSII, *Φ*_PSII_ = (*F*_m_′ − *F*_s_)/*F*_m_′.

### Transmission electron microscopy

The growth status of apple calli and severity of *B. dothidea* infection were observed via TEM. Then, samples of 1 mm were excised from calli infected with *B. dothidea* for 4 days. The surface of each sample was covered with a thin layer of agarose gel to prevent calli dispersion. Then, the samples were immediately submerged in glutaraldehyde (4%) for at least 24 hours at 4°C. They were rinsed in phosphate buffer (0.1 M) five times for 20 minutes each time and then immersed in an osmium tetroxide solution (0.1%) for 4.5 hours. The samples were again rinsed in phosphate buffer (0.1 M) five times for 20 minutes each time. Then, the calli samples were dehydrated in a graded ethanol series, immersed in epoxy propane twice for 1 hour each time, and embedded in pure resin. Ultrathin sections were cut using an ultramicrotome and observed using a JEM-1400 Plus TEM.

### RNA extraction and quantitative real-time PCR analysis

Total RNA was isolated using an RNAprep Pure Plant Kit (TIANGEN, China) in accordance with the manufacturer’s instructions. First-strand cDNA was synthesized using a First Strand cDNA Synthesis Kit (Thermo Fisher Scientific, USA) based on the manufacturer’s instructions. The CFX96TM Real-time Detection System (Bio-Rad, USA) was used to perform qRT–PCR. The following program was used: predenaturation at 98°C for 5 minutes; 40 cycles of 98°C for 15 seconds and then 60°C for 30 seconds; and a final melt cycle from 60 to 98°C. The *Malus* × *domestica actin* gene (*MdActin*, GenBank ID XM029089583.1) was used as the housekeeping control. Twelve photosynthesis-related genes were selected from the Photosynthesis KEGG pathway (KEGG ID mdm00195) based on the 197 genes shared by cluster 3 and the brown module in WGCNA or the 99 genes shared by cluster 4 and the brown module in WGCNA. The primers used in the qRT–PCR analyses are shown in Supplementary Data Table S12.

### Gene cloning, vector construction, and genetic transformation

Full-length coding sequences or fragments of *MdS6PDH1* (Gene ID XP_028950895.1) were cloned from apple via PCR. The primers used are shown in Supplementary Data Table S12. For silencing *MdS6PDH1* in calli, an *MdS6PDH1* fragment from 85 to 309 bp was used for RNAi vector construction. This fragment was transferred to pART27 vector to generate the RNAi transformation vector. To obtain *MdS6PDH1* overexpression calli, the full-length open reading frame (ORF) of *MdS6PDH1* was inserted into a pPZP211 vector under the control of the cauliflower mosaic virus (CaMV) 35S promoter to generate the overexpression transformation vector. The recombined constructs were transformed into *Agrobacterium tumefaciens* LBA4404. Transgenic calli were obtained based on previously described methods [10].

### Statistical analysis

The experiments were performed with at least three independent replicates. The variability of the samples is indicated by the mean ± standard error of three repetitions. Statistical significance between different measurements was determined using Tukey’s HSD test with SPSS statistics software version 19 (IBM, USA).

## Acknowledgements

This work was financially supported by grants from the Natural Science Foundation of Shandong Province (Grant No. ZR2020QC147), the Agricultural Variety Improvement Project of Shandong Province (Grant No. 2019LZGC007), the Agricultural Major Applied Technology Innovation Project of Shandong Province (2018), the Agricultural Scientific and Technological Innovation Project of Shandong Academy of Agricultural Sciences (CXGC2021A03) and the Earmarked Fund for China Agriculture Research System (CARS-27).

## Author contributions

Che.W. and L.L. conceived the project and designed the experiments. X.H. and H.M. performed most of the experiments and acquired and analyzed the data with the assistance of H.W., P.H., Y.C., S.W., and Chu.W. Che.W. and X.H. performed the bioinformatics analysis and wrote the article.

## Data availability

The data that support the findings of this study are available from the corresponding author upon reasonable request.

## Conflict of interest

The authors declare no competing financial interests.

## Supplementary data

Supplementary data is available at *Horticulture Research * online.

## Supplementary Material

Web_Material_uhac115
